# Lectures on Clinical Medicine, Delivered at the Philadelphia Medical Institute

**Published:** 1838-07-04

**Authors:** W. W. Gerhard

**Affiliations:** Physician to the Philadelphia Hospital, &c.


### CLINICAL LECTURES.

### LECTURES ON CLINICAL MEDICINE, *delivered at the Philadelphia Medical Institute, by* W. W. GERHARD, M. D., *Physician to the Philadelphia Hospital*, &c.

### DELIRIUM TREMENS.

(Concluded from page 216.)

After you have, for some time, attempted to tranquillize your patient with opium and brandy, and he still remains restless, examine, if there be not some cause of it, other than the delirium tremens. This may be inflammation of the membranes of the brain, congestion of that organ, or an irritable condition of the alimentary canal. These symptoms may be relieved by cupping and an injection, and then with opium and assafœtida, you will put the patient to sleep. If he still remain awake, you may cautiously increase the quantity of laudanum to a drachm every two or three hours, but not beyond this amount, and try other remedies, as camphor. If the nervous symptoms yet persist, you will gain a great deal by giving an opiate injection, containing about thirty drops of laudanum, and repeat it after the lapse of several hours. But I am not disposed to increase the opium to those excessive doses which have been given in the management of this disease.

We had, some time since, a case of delirium tremens, complicated with pneumonia. In these cases, you must give opium in small doses, as large quantities of it would suddenly check the secretion into the bronchi; you may substitute for it brandy and carbonate of ammonia. This last remedy is excellent, under these circumstances, tending to increase the expectoration; it proved most serviceable to a patient who was under my care a few days ago. You may give five or ten grains of it, every two hours, and add to it, if the patient sinks, brandy and punch. Blisters over the inflamed lung are here of two-fold advantage—aiding in tranquillizing the patient, and counteracting the pulmonary disease, better than depletion. You must remember that pneumonia, in these drunkards, tends singularly to run into gangrene, and will not bear very copious depletion. If you have convulsions and congestions, free cupping will generally arrest them, combined with cold applications to the head, bladders of ice, bags of cold water, or an evaporating lotion.

These remedies will generally prove sufficient; but, if the disease reaches the third or muttering stage, there is great danger, and only one remedy can save the patient. This is a blister to the back of the neck, or to the occiput. It is inappropriate at an early period, when there is thirst and fever; but, in the last stage, it is of capital importance, and will often preserve the life of the patient. It is rarely to be applied over the whole head.

Chronic cases of delirium tremens are very difficult to cure, generally occurring in broken-down constitutions. If the patient be confined, he cannot be cured; he must walk about, and have free exercise. In addition to this, cold to the head, tonics, capsicum, and a good diet will often cure these cases. Blisters frequently applied behind the ears, are also here very serviceable.

Connected with this subject, there is only one other point to be noticed—the insanity following drunkenness. In this variety there is great restlessness, discrepancy, and incoherence of ideas, in fact, soon afterwards, complete dementia. It is to be managed chiefly by moral remedies.

This slight sketch will be useful to you, as a guide to the management of an affection, of lamentable frequency in every part of our country.

---

### CERTAIN DISEASES OF THE SPINAL MARROW.

*Friday, June 15th.*—I shall, to-day, make a few remarks on the subject of diseases of the spine, which are suggested by the case of a man, who furnishes a very striking illustration of this class of affections. He was admitted this morning, but was in the hospital, three years since, under my care, for an affection of the spinal marrow, the symptoms of which were pain down the spine, extending thence, along the course of the sciatic nerves, more on the left side than the right, accompanied by a slight sense of weakness, across the back and in the limbs, not, however, amounting to paralysis. This complaint had begun two years before the man entered the hospital, so that the affection dates its origin to a period five years antecedent to the present time. When the man was first in the hospital, he was treated on a systematic plan, kept up with much perseverance. He was cupped down the spine, and along the course of the sciatic nerve, from which considerable advantage was derived. Long strips of blister were applied, also in the same direction; and counter-irritation was afterwards made by the moxa, an excellent and not very painful remedy for this purpose. A single moxa was applied, every day, in such a manner as to produce a superficial ulcer, so that there were always three or four of these points of counter-irritation. The man went out relieved, and was directed to continue the use of the moxa; this he did not, however, do, and of course the remedy had not a fair chance of success, although the man was, certainly, vastly relieved by its partial application. The patient is obliged to return to the hospital, from the failure of his strength. He now, however, no longer presents symptoms of an affection of the medulla spinalis alone, but of the bones as well as the medulla; the disease having probably originated in the former texture, but, as it was unaccompanied by any distortion, it was impossible to ascertain whether the spinal chord was the original seat of the affection, or not. There is now a tumor in the region of the sacrum, and an obvious prominence of the lumbar vertebræ; and the man now walks like a paraplegic patient, supporting the upper part of his body by leaning forwards, and resting his hands upon his knees. Having lately lectured upon the subject of apoplexy, we may now find it interesting to trace paraplegia in contrast with hemiplegia, or the paralysis depending on the spinal marrow, in contrast with that depending on the brain. Hemiplegia may be the result of simple neuralgia, and is therefore not an infallible indication of cerebral disease; but there are, in such cases, numerous other signs of the nervous character of the disorder, which will prevent you from confounding it with the cerebral paralysis to which I am now alluding.

To study paraplegia to most advantage, it will be best for us to examine the symptoms of injury to the spinal marrow, resulting from fracture of the vertebræ, in which there is complete loss of motion, and of the powers of the bladder and the rectum. The effects of the excessive paraplegia, resulting from an injury of the spinal marrow, are most strongly manifested on the organs contained within the pelvis; and, in consequence of the abstraction of nervous sensibility from the parts, the patient dies from over distention of the bladder with urine or pus, or else gives way under the irritation attending the sloughing caused by pressure in the region of the sacrum and trochanters. The case under notice has not advanced, by any means, to the condition I have just described. The patient has still some control over the powers of locomotion; but, you saw, this morning, when I directed him to walk, that he did not hobble as in hemiplegia, but bent his body forwards, to fortify the muscles of his back, the muscles most difficult to sustain in the act of walking, and to alter his centre of gravity.

The next point to be considered is the loss of nutrition, which has taken place in the lower extremities, particularly in the left leg, which is thinner than the right. This is, of course, owing to the wasting away of the muscles, from long disuse. The change in the sensation of the limbs, is also worthy of notice: in the legs there is a feeling of pricking, or of formication, or of numbness, or the sensation which is caused when the limb is said to be asleep. This arises from the pressure on the nerves of the limb. Spasms may occur in diseases of the spinal marrow, but are not usual when they are chronic.

Let us consider the diagnosis, prognosis, and treatment of this case. First, the diagnosis.—When the man came into the hospital three years ago, we knew that he had an affection of the spine, but we could not ascertain whether the vertebræ were in a carious condition, or not. The diagnosis of disease of the vertebræ cannot be made out, with certainty, at an early period of the affection. But there are usually, as in the present instance, some circumstances, that assist very much in forming a diagnosis, although it can be only an approximate one. When the lymphatic glands of the neck and other parts are in a swollen condition, or if there be other indications of scrofulous diathesis, such as tubercles in the lungs, or that peculiar physiognomy which is so often observed in such patients, we may infer that the disease of the spine is of a secondary character, and dependent on a constitutional taint, developing itself in the bodies of the vertebræ.

The organic diseases of the spinal marrow are numerous, and some of them are with difficulty distinguished from the secondary alterations, just alluded to. Apoplexy of the substance of the medulla is a rare disease—so rare, that I have never seen a dissection of it; but membranous apoplexy, in which the blood is poured into the sheath of the medulla, I have several times witnessed; in both these varieties, the disease is sudden in its onset, and is attended with paraplegia. Where the effusion of blood occurs into the membranes, the paralysis is more extensive, and gradually tends to ascend from the lower extremities, the nerves of which are supplied from the lower part of the spine, towards the upper parts of the body; death follows as soon as the muscles of respiration are palsied. In the apoplexy of the nervous substance, the paralysis is more fixed, and, if not complete, the patient may recover. Neither of these varieties can well be confounded with cerebral hæmorrhage, in which there is nearly, if not quite always, palsy of the side of the body only. In the case of the patient just admitted, as well as in most others who are affected with spinal paralysis, the intelligence is remarkably clear, and more bright than usual, which is another point of distinction between the affections of the spinal marrow and those of the brain. I have already stated, that we can distinguish, with great difficulty, chronic inflammation of the medulla, from the effects of scrofulous disease of its bony covering.

The prognosis, in this case, is not difficult. From the fact of its dating its origin to five years since—from its having been partially arrested, and now returning with aggravated symptoms, there is, we fear, but one termination for it—it will probably end in death. But before the disease of the vertebræ is established, if the early symptoms are combated, there is a tolerable chance of success; now that evident distortion has taken place, indicating extensive bony disorganization, the affection is almost necessarily fatal.

The treatment, proper in this case, applies to all chronic inflammations of the spinal marrow. They are to be managed on the same principles as those of the brain; the treatment is, however, more local in its character. Purging should be employed with a view to act upon the local disease, as a revulsive; instead of diminishing, it rather keeps up the general health. Rest is essential: hence, as in cerebral apoplexy, I would avoid stimulating the organ with strychnine, or remedies of the sort, unless in the very chronic stages; keep it perfectly quiet, by placing the patient on his back, and retaining him there, rigorously. If the symptoms are tolerably active, cupping must be freely employed; but only in a moderate way, if the affection be altogether chronic. After local depletion by cups and leeches, remedies of a more permanent character must be used. There are several which may be tried, and a preference given to that which is found to answer best, in the particular case. From blisters I have derived but very slight advantage. Moxas I have found decidedly the most useful, though I cannot say that they are always permanently serviceable. They have several advantages;—they produce the same good effects as the caustic potash, or setons, and are much more moderate in their action, and less irksome to the patient; they likewise, from the pain they produce, stimulate the nerves in the least injurious way, being thus of double service—as a counter-irritant and an excitant. They are to be applied frequently, say one every day, or every other day, and on half-a-dozen different places, which may thus be kept sore; their application being continued, until they produce vesication, but not deep ulceration. In this case moxas were used for two or three weeks; and, in other cases, I have used them for months. If they give intense pain, they may be given up, and other remedies tried, as the caustic, setons, and blisters.

Acute inflammation of the spinal marrow is an affection of rare occurrence. The most unequivocal case I ever saw which terminated fatally, took place at the Children's Hospital at Paris, in a child six or seven years of age. The little girl was very intelligent, and could give a very good account of herself. She had received no injury, and there was no obvious cause of the disease, but the constitutional tendency to it, which was evident from another sister soon after entering with nearly similar symptoms. She entered the hospital, after having been ill for three days. At this time there was stiffness and flexion of the limbs of the upper and lower extremities, which could not be extended without great pain, and this was the only uneasiness felt; when the limbs were touched she did not complain. This contraction continued to increase till death, and was always greatest in the upper extremities, the arms being bent to an acute angle, at the elbow. Upon examination after death, the upper portion of the spinal marrow was found in a softened condition, particularly, just below the crossing of the corpora-pyramidalia. The softening involved both the anterior and posterior portions of the spinal column, thus accounting for the affection of the nerves of motion and sensation according to the theory, at present generally received, which, however, some recent experiments appear to call in question.

The general symptoms, then, of acute inflammation of the spinal marrow, are numbness, pain, stiffness, and rigidity of the muscles, supplied from nerves coming off below the part affected, and if the inflammation is towards the upper portion of the spinal marrow, the contraction is more obvious, in the upper than the lower extremities. If in the case to which I have alluded, the pain had been unaccompanied with contraction, I should not have deemed it a necessary symptom. The functions of the alimentary canal were not altered in a remarkable manner. The appetite was good, and the powers of urining not impaired, although this cannot be generally the case. The integrity of the cerebral functions, in this patient, sufficiently showed the spinal marrow to be the origin of the symptoms.

The treatment must depend upon the activity of the affection, and the period at which you are called to it. The case of the little girl was not so actively managed in the French hospital, as it would have been by our practitioners. There the treatment was confined to some remedies of a local character; but with us, it would have been looked upon as an affection of a most severe grade, (for the patient inevitably dies, as soon as the disease extends to the neck and the functions of respiration become affected,) and we should have treated it accordingly. A patient of a strong constitution, I should bleed freely, and follow the bleeding up by cupping over the part affected, to an almost indefinite extent. If the patient be feeble, the depletory treatment must be somewhat modified, and confined to leeching. After this plan has been pursued for several days, it is proper to attempt to moderate the local symptoms, by blisters, moxas and purging, the latter of which, however, is to be looked on as a mere adjuvant. This affection is not a rare one in children; like most diseases, however, it assumes something of an epidemic character, and you will have, at certain periods, a number of cases together, and may afterwards not meet with one for several years.

There are a number of affections of the spinal marrow, into which I shall not enter in this course—some of a very tangible character, and others, protean in form and nature. Within a few years past, it has been the fashion to ascribe to affections of the spinal marrow, an almost endless variety of symptoms—some with propriety, but others on very inadequate grounds. This subject was investigated, some years since, by Tate and Teale, of Yorkshire; and the results of their researches attracted to it considerable attention. Three or four years ago, these complaints were more common, certainly, than at present, and, hence, some of the interest with which they were regarded, has subsided.

Under the term, spinal irritation, which has also been given to the more indefinite class of affections, I am disposed to include two undoubted varieties of disease; first, all cases of neuralgia, originating in the spinal column, and extending along the course of one or more nerves, and in which there is pain on pressure over the corresponding vertebræ. If the pain be greater over the vertebræ, than in the muscles affected; if you have, for instance, pain over the scapula, and upon pressing the spine, you find it more tender than in the muscles of the scapular region, you may at once conclude that you have neuralgic rheumatism. Secondly, I use the term, also, in those cases of more obscurity, in which you have pain in a circumscribed spot, at the anterior part of the chest, near the heart, or sometimes, although rarely, at the same point of the right side. In these cases, if you find a tender spot in the vertebræ, corresponding with this cardiac pain, it is fair to infer the existence of spinal irritation. The diagnosis becomes more clear, if you have also pain on pressure, along the course of the intercostal nerves; although such an extension is by no means necessary. Many are disposed to account for other functional disorders, by tracing them to spinal irritation, cases of palpitation of the heart, in which there is no anemia, and where there is pain on pressure over the vertebræ, may be referred to this cause. If the latter symptom be wanting, you can decide only by the results of treatment.

Affections of the alimentary canal are also traced to the same cause. You may recollect the prevalence of dyspepsia all over the country, some years ago, when lawyers, clergymen, and all who pursued sedentary occupations, as well as some who led a more active life, were uttering universal complaint on this subject. Much of this undoubtedly depended upon spinal irritation: how far the spinal marrow was connected with the symptoms, was to be known from the evidence of pain on pressure, or by the success of the after treatment. Disorders of the lower bowels are never ascribed to spinal irritation.

Some are disposed to deride the very name of spinal irritation, as applied to the last mentioned disorders; but I think it may still, with propriety, be given to certain groups of symptoms, for want of a better term by which to designate them. Although they are not now met with, so often as they occurred a few years ago, they still present themselves in sufficient numbers to claim a fair share of your attention.

There is one other affection, in which the spinal marrow sometimes plays an important part. This is acute articular rheumatism, in which we find another element in addition to mere neuralgia. In articular local inflammations, if you can detect pain on pressure along the spine, and if the local pain is at the same time increased, cups along the spine, as recommended and successfully used by Dr. Mitchell, of this city, will often prove of signal service.

The diagnosis of the various disorders, dependent on irritation of the spinal marrow, is not a little difficult; and, from the kind of enthusiasm with which the researches of Teale and Griffin were received, ludicrous mistakes are sometimes made, in attributing to this cause, important functional disturbances, in which the spinal marrow is not at all affected, or only in a slight and secondary manner. I shall point out some of the modes of discrimination, in these cases. In affections of the chest, of the lungs or heart, physical examination may be resorted to, to determine all points of doubt. In disorders of the stomach, the best test of the presence of gastritis is afforded by the effects of food, which proves irritant as soon as taken, in chronic gastritis; whereas, in neuralgia, although the ordinary aliment may occasion some inconvenience, slight stimulants, as wine, are both salutary and proper.

The pathology of the class of affections which we are examining, is unknown. The probability is immense, that there is no pathological alteration in the organ, but that the disturbance is altogether functional. Besides, the disorder generally disappears so long before death, as not to allow us to detect any morbid alteration, that may have previously existed. If to this we add the great difficulty attending the pathological examinations of the spinal marrow, we can readily account for our ignorance on the subject. Physicians, however, generally treat these affections, as if they were known to be inflammatory, by local depletion, and, afterwards, counter-irritation by blisters, tartar emetic, and croton oil. The tartar emetic is a good remedy for this purpose, but a painful one; the croton oil is less painful; and blisters cease to give much annoyance, after one or two applications. This mode of practice is found by experience, to be the most successful, and in our ignorance of the precise nature of the lesions, we cannot do better than pursue it.

I had lately a marked case of obvious functional disorder of the heart, the symptoms of which disappeared, after one or two blisterings to the back. Another similar case, was that of a woman in the hospital of the almshouse, who had been for a long time unavailingly treated for gastritis, and severe pain in the region of the stomach, which were relieved at once by one or two cuppings to the spine. About the same time, a friend of mine, a young lawyer, of high promise, offered a well-marked example of a similar affection. He had been treated for it, with little success, by a country physician of skill and experience, when he applied to Professor Jackson, who, just at this time, had become acquainted with the then novel subject of spinal irritation, and, a few leeches and counter-irritation to the spine, directed by him, effectually relieved the symptoms, which did not return. This simple treatment will often succeed, without resorting to tartar emetic or other irritating remedies.

In cases of organic diseases of the heart, complicated with spinal irritation, Dr. Marshall, who has written an interesting treatise on this subject, recommends the direction of our therapeutics to the cure of the latter. I have seen many such cases, and have often succeeded with great facility, in so far modifying the symptoms, that they have ceased to annoy the patient; for if, in these cases, you remove the functional disorder, the organic lesion often proves of little injury to the comfort of the patient. A gentleman now residing in Washington, consulted me a few months ago, in an analogous case, in which there was considerable valvular disease, with extreme functional disorder of the heart. I succeeded in removing the latter symptoms, which were in this instance, treated without irritants to the spine, and the patient is scarcely conscious of the heart disease. Now, in such cases, the local remedies addressed to the spine, often prove of extraordinary advantage. In diseases of the lungs, less success is to be anticipated, than in those of the heart or stomach. Whenever you find irritation distinctly extending from the spine to the viscus affected, the success of the spinal treatment may be looked upon as almost certain, and relief will often be almost instantaneous. Last summer we had several cases, illustrative of the good effects of this treatment, and, although none offer themselves at this time, we may look for their not very distant return.

These brief remarks will prove sufficient for the purpose for which they are intended—to call your attention to an important and very troublesome affection. For fuller details on the subject, which are not now demanded, I refer you to several capital treatises, perhaps a little ultra, in some of the points which they urge. They are those of Tate; of Teale, on Hysteria; of the Messrs. Griffin, of Ireland, a more complete and distinct work, in which the protean forms and transient character of these affections, are amply discussed and illustrated by cases; that of Marshall, of Scotland, and, afterwards, of the North of England, (not Marshall Hall,) a valuable work, which has been republished in this country, and from which you can extract much that is excellent, allowing for some exaggerations of views and facts. I say exaggerations, for others have certainly not been so successful, in the management of neuralgic affections, as this author seems to have been.

Should many other nervous affections present themselves during the course of the summer, I shall take them up; but it is probable that our attention will be occupied with more acute and violent affections. Besides, the excellent series of lectures which were given by Dr. Jackson, upon this subject, during the past winter, (which you will find in the earlier numbers of the Medical Examiner,) renders it less necessary for me to enlarge upon the subject of these diseases.

---

### INFLAMMATIONS OF THE MUCOUS MEMBRANES.

*Tuesday, May 29th.*—You are aware that I have devoted a considerable portion of the early part of this course, to the study of diseases of the serous membranes. My reason for this order was, in part, derived from the extraordinary prevalence of these affections; but it arose, in a still greater degree, from the aid it will afford you in the study of disease. The serous tissues are so closely connected with various viscera, of the highest importance to life, that we are led, as it were, by a natural connection, to pass from the study of the inflammations and diseases of the investing membrane, to those of the parenchyma itself. We thus group together clusters of diseases, and acquire those habits of close analysis and careful comparison of symptoms, upon which diagnosis is based. You have, in this manner, been led to study apoplexy and softening of the brain, after commencing with meningitis, in which the signs of serous inflammations in general, are so much blended with the symptoms peculiar to cerebral disease. You had been already prepared for the study of meningitis, by the analysis of the symptoms observed in the inflammations of serous membranes, which are of a less complex character—such as the pleura or peritoneum. You have also heard, that the symptoms of these inflammations arise to a considerable proportion, from violence or injury done to the part, whether this injury result from blows or other external causes, or from perforation caused by disease of the parenchyma.

These accidental and, as it were, artificial serous inflammations, present to you the most simple form of diseased action. The serous tissue is remarkably well fitted for the examination of every stage of this process; it is so transparent, that every new vascular ramification can be traced, creeping over its surface, and then, as all these membranes form perfect sacs from which there is no outlet, the products of inflammation are retained, and each step in the process is distinctly known. You see, therefore, how very useful it has been for you, to commence the study of pathology, by its simplest and most beautiful phenomena.

With the gradual progress of your investigations, and the habits of observing disease which you have now acquired, it will be easy for you to enter upon the study of the diseases of the mucous tissue, and, from thence, to pass to the various affections with which they are most closely connected. But you must not expect to meet with the same simplicity and regularity of phenomena, that you have seen in the serous membranes. The structure of the mucous lining of the viscera is more complex,—it varies in some degree in different organs, and, again, is still more complicated from the numerous follicles disseminated throughout it, which form, in some respects, independent organs, and are subject to diseases of a different character from the membrane itself.

The secretions of the various parts of the mucous membranes are as different as their anatomical structure, and, in a pathological state, the diversity is at least as great as in the normal condition. Now, these secretions offer one important point of difference from those of the serous membranes, besides their complexity; they are excreted, in many cases of diseases, and thus furnish us signs of considerable importance for the diagnosis. These varieties depend, partly upon an augmentation in the natural secretions, and partly upon new products, formed by the more violent grades of inflammation. In the serous tissues, on the other hand, there are no secreting follicles; the moisture upon their surface is composed simply of serum, and, when inflammation occurs, you find its products are unmixed with any peculiar secretion from the membrane itself.

The secretions from an inflamed mucous membrane usually pass through the following changes, which will be found to vary greatly in the different portions of this tissue. Let us first examine the secretions of an inflamed Schneiderian membrane, which is partly within sight, and, therefore, in a situation the most proper for observation. The first appearance of coryza is an extreme dryness of this membrane; in the natural state there is mucus sufficient to protect the tissue from the irritating effects of the external air, to which it is constantly exposed; but, in inflammation, the sensation of dryness amounts almost to pain. The diminution in the quantity of mucus is the best indication of the passage from that period, in which the vessels are stimulated, to that in which they throw out more mucus than in the healthy state—from mere irritation, to the real inflammatory action. But the mucous tissue does not long remain more dry than usual; it begins again to secrete a liquid, which consists of little else than the normal mucus, augmented in quantity; the disease may terminate here, and, in a large proportion of cases of coryza, does not extend further. But, if it advance, we find a gradual passage of the secretion through changes which are very analogous to those occurring in the serous tissues. The mucus becomes opaque, from the admixture of lymph, and, if the affection advance still further, assumes a yellow tint, from the admixture of pus. In certain very intense stages of inflammation, pure pus is secreted.

A peculiarity of the inflammations of mucous coats is, that they rarely adhere, or become completely obliterated, as in serous inflammations, which partly arises from the constant passage of liquid over the inflamed surfaces, and partly from the admixture of mucus with the lymph, which constitutes the basis of all false membranes.

These secretions are those which may be termed natural, and occur almost in all mucous inflammations; but there are many others, which are accidental, and are present only in very high grades of inflammation. Blood is often effused in dysentery, in very considerable quantities, pure and unmixed. In bronchitis and pneumonia it is also secreted, but, in less quantity, and more intimately mingled with the mucus and pus. The effusion of blood is, in part, a natural means of relieving the inflammation of the mucous tissue; it is very rare in the inflammations of the serous membranes, where the natural method of cure is much more easy and regular, and consists in the secretion of serum and lymph, and afterwards in adhesions of the two surfaces of the membrane. If the blood be only in moderate quantity, we are not accustomed to regard it as a symptom of danger, or one that should be interrupted by very energetic means. But when the local inflammation has passed to the stage of ulceration, a large discharge of blood becomes a sign of much gravity, and should, if possible, be arrested.

Another morbid discharge occurring from mucous surfaces is still more rare, that is, the gangrenous discharge arising from sloughs of the mucous membranes, which are as frequent in their severe inflammation, as they are rare in the serous tissue. A very fœtid discharge will, however, sometimes take place, without sloughing, from the mere change of secretion from the diseased surface; it is always attended with danger, and is extremely difficult to remove.

These various secretions pass towards the exterior, and are discharged from the body; we can thus, to a great extent, ascertain the degree of the inflammation by means of the discharge. Although we have no means of estimating the extent and progress of the lesions, equally precise with those afforded by physical examination in most diseases of the serous membranes, we yet possess an important diagnosis in the alterations of the secretions. If the inflammation be seated near the point of termination of the mucous membrane, these morbid secretions are nearly mixed with the healthy secretions of the part, and the diagnosis is consequently more easy; but when it is remote, the nature of the disease can only be known by a more difficult and more careful estimate of probabilities.

The follicles contained in the mucous membranes may be either wholly exempt from their inflammations, or may participate in them to a very slight degree. Thus, in many cases of dysentery, the follicles are scarcely discerned, and in some chronic varieties of this disorder, the follicles are less developed than in the natural state, they may even totally disappear. Most commonly, the follicles of the large intestine are inflamed at the same time with its mucous coat; but those of the small intestine and stomach seem to possess an independent vitality, and are diseased from causes not connected with the mucous coat. In the trachea and larynx, both follicles and membrane are usually inflamed at the same time, although in very various degrees; for some diseases, such as small-pox, often cause inflammation of the follicular structure, whilst in others, which are of a less specific nature, it is usually confined to the membrane. Secondary inflammations occur more frequently in the follicles than in the membrane, which furnishes you with another proof of their difference of structure and of functions. These inflammations are sometimes almost specific, and differ in their progress and termination from those which are confined to the local change of structure. The tuberculous disease, in a very large proportion of subjects, extends to the follicles of the alimentary canal, and shows a singular preference for those which are situated in certain parts of the mucous tissue, the glands of Peyer, and in the isolated follicles of the small intestine, these tuberculous deposits are especially numerous; they are rather less frequent in the large intestine, and are comparatively rare in the follicles of other portions of the mucous membranes. Tubercles are thus less intimately connected with the mucous than the serous membranes, they are not deposited in the thickness of the tissue, but are entirely contained on its fore surface, or rather in that portion of its surface which lines the follicular sacs. This mode of secretion of the tuberculous matter has induced Dr. Carswell to consider tubercles as the product of the mucous tissue in all cases. This is, however, an opinion which you know to be incorrect, for in no part of the body are tubercles more perfectly developed than in the serous membranes; you have traced them in their earliest stages where a very remote semi-opaque point was the only proof of their tuberculous nature, to the fully formed, yellow, hard tubercle. But a few days ago, I demonstrated the formation of tubercle in the glands of Peyer; you then saw that it was contained in the centre of the follicle, and presented the form of a semi-transparent secretion which had scarcely assumed the solid form.

Ulceration is a very frequent, almost a necessary consequence of the diseases of the follicles. In the tuberculous diseases, ulceration follows the softening, and consequent elimination of the tubercles, and is very analogous to the same process occurring in the lungs. In ordinary inflammation, an ulcer may succeed a deposite of pus, which accumulates in the centre of the follicle, and is similar to other abscesses; in other cases, a slough forms, in the cellular tissue beneath the gland, and the mucous membrane covering it, is afterwards destroyed by an extension of the mortification; in a third variety, ulceration occurs as a direct consequence of inflammation, and is preceded by neither slough nor abscess. The appearance of these ulcers is various; but they are readily traced throughout their various stages, and referred to one single lesion. After the inflammation surrounding the ulcer declines, cicatrization takes place by the gradual sinking of the edges of the ulcer, and the conversion of its bottom into a smooth, shining, cellular tissue. After the ulcers are entirely healed the cicatrix remains, and the mucous membrane retains ever after, a shining, bluish colour; it is much smoother than the adjoining coat, and is deprived of its villi. The puckered, wrinkled aspect of cicatrices is compared to that which succeeds the largest ulcers, and is similar to the appearance of the newly formed skin, which is constantly formed after the cure of external ulcers. These cicatrices are extremely common, and prove how very readily a mucous membrane will pass through all the stages of inflammation, and will finally be converted into a tissue scarcely differing from its original structure.

Ulceration is as frequent in inflammations of the mucous membranes as adhesion is rare; the latter process is partly prevented by the constant passage of the secreted liquids over the diseased surface, which prevents them from remaining in contact; and, in part, by the difficulty with which a mucous membrane secretes lymph. The adhesions which sometimes occur are confined to those portions of these membranes, which are remote from the exterior, and which do not serve as the channel by which secretions from more distant parts reach the surface. You will scarcely meet with them, except in the minute ramifications of the bronchial tubes, where they occur after violent pneumonia and bronchitis: the smaller bronchial tubes are the more subject to this change, from their tissue resembling very closely that of the serous membranes.

We shall next examine the changes which take place in the mucous membranes, during the progress of acute and chronic inflammations, or, rather, I shall content myself with merely pointing them out, and will refer you for more complete developments, to my lectures on pathological anatomy. The first alteration is the colour; in the earlier stages of inflammation the vessels become more numerous, but are not so abundant nor distinct as in the serous membranes. The redness is bright, of an arterial tint, and is disposed in dots and minute vascular branches; you must recollect, however, that although this sort of dotted redness is a very good proof of inflammation, its absence does not show that none was present, for in great part it may disappear after death. To study the true character of inflammatory redness, you should examine the tracheal or bronchial tubes, or in fatal cases of dysentery, the large intestine; these are the situations where inflammation is most common, and in which opportunities for examination after death most frequently occur. A very good opportunity is also presented in the gastritis which follows poisoning by arsenic and corrosive sublimate. I have seen instances of the effects of both these poisons, and found the whole mucous coat of the stomach of a bright, punctuated redness. If the inflammation be very intense, the redness may assume a darker tint—purple, or almost black, especially in certain cases of dysentery, where the whole of the mucous membrane, which is not removed by sloughing or ulceration, is of this livid colour. Now, this alteration depends mainly upon the stagnation of blood in the vessels, and is closely allied to gangrene. In chronic inflammation the colour of the membrane varies from red to a dark lead or slate colour; the latter usually predominates.

The alterations of thickness and consistence of inflamed mucous coats are various; as a general rule, they may be reduced to a few simple points. In acute inflammations these membranes are thickened and softened; in chronic, their consistence is increased, but if the inflammation continue, they are still softened, but in irregular portions. These alterations of consistence also depend somewhat upon the part of the membrane; thus in chronic inflammations of the stomach, the membrane is thickened and hardened—the same thing often occurs in the bronchial tubes; it is thinned in the intestinal canal much more frequently than thickened, and its consistence is often diminished. These varieties must all depend upon some regular laws, probably on the fluids secreted by the membranes, which vary in their chemical properties, in different parts of the intestinal canal. You might render an essential service to the science, by following out the researches commenced a few years ago in Paris, by Dr. Bowditch, of Boston; and, by carefully analyzing the fluids formed in various parts of the mucous membranes, you might greatly facilitate the diagnosis of their diseases.

In examining the diseases of the serous membranes, I stated that certain general symptoms attended the acute inflammations of this tissue. These symptoms consist in chill, fever, sweating, and acute lancinating pain; the latter symptom is, however, by no means invariable. Now you see that the signs of the local inflammations are followed by secondary excitement of the circulation; but there is little disturbance of the functions of the brain or the digestive organs. The disease is readily localized, and is sufficiently uncomplicated for us to distinguish from analogous affections. In inflammation of the mucous membranes, on the other hand, the local symptoms, as pain or other uneasiness, are generally obscure, and sometimes can only be ascertained by carefully examining the various parts of this tissue. Fever exists both in the inflammations of the serous and mucous membranes, but it is less active in the latter than in the former case, and is attended with more disturbance of the functions of nutrition, and of sensation. That is, all inflammations of mucous membranes possess a more extensive action upon other, and distant organs of the body, than those of serous membranes; they are, in some sort, concealed in the midst of a complication of symptoms, which it is very difficult to unravel. You may trace this complexity of symptoms in nearly every disease of a mucous membrane; bronchitis, diarrhœa, even gonorrhœa affect the intelligence and appetite of the patient, as well as the functions of the membrane primitively affected. These complicated reactions become, of course, most manifest in chronic diseases, and when you meet with a chronic inflammation of the rectum or urethra, you will often be surprised at the difficulty you find, in discovering the original disorder; the patient will even call your attention to nearly every symptom, before alluding to those immediately connected with the part affected. Hence you should never forget the practical fact of the diffusive sympathy of the mucous membranes.

Serous membranes possess no appreciable function, other than that of enclosing an organ, but mucous membranes are the seat of many important functions. Now, the functional disturbance in serous inflammations is limited to the impediment to the motions of the subjacent organ, but the mucous membranes present a much more immediate, and, as it were, peculiar functional disorder. We therefore regard nausea, anorexia, vomiting, and difficult digestion, as the symptoms of disorders of the stomach; cough, and altered secretions of those of the bronchial tubes; while various forms of diarrhœa are indicative of inflammations of the intestines. These symptoms you may study more at your leisure, when we inquire into the history of individual diseases.

I have already alluded to the secondary inflammations of the mucous membranes; these are extremely frequent, and attack both the follicles and the membranes itself. They are to be found in every part of this tissue, and result both from local and constitutional causes. In phthisis you have examples of both varieties; the irritating secretions from the bronchi and the tuberculous cavities, pass over the trachea and larynx, and cause ulceration of their follicles and inflammation of the membrane; while in the intestine, the follicles inflame from the deposit of tuberculous matter in them; that is, from the action of the same general cause which gives rise to the deposit of tubercles, in the lungs and other organs. In typhoid fever we have a follicular disease, arising also from a general cause, which shows itself in the follicles, but does not, strictly speaking, result from their inflammation. Slighter forms of these secondary inflammations occur in all diseases attended with much vascular excitement, for fever, sooner or later, will infallibly cause a disorder of the mucous coat.

There are forms of inflammation of the mucous membranes, which are more complex. In the slighter cases of dysentery you find a disease of the large intestine, which is strictly local; but, in the more severe forms, it would appear that the disease approaches very nearly to the character of a general affection, and that the disease of the mucous coat is merely one part of a complicated series of lesions. Thus you will find that most epidemics of dysentery are characterized by an extraordinary developement of nervous symptoms, and that the inflammation of the colon is not always in proportion to the gravity of the disease.

You are aware of the important part attributed to the mucous membranes in fever. Now, although in remittent and intermittent fevers, it is perfectly true, that the stomach rarely escapes, I am far from concluding that the essence of the disease consists in gastritis. On the contrary, you will find that this inflammation varies very much in importance, and at times, its symptoms can scarcely be recognised. Still, although not precisely the point of origin of the phenomena of fever, it is a very important complication and should be watched with extreme care. Broussais has done much for the profession notwithstanding his exaggerated views, and the practice which he has so earnestly recommended, will be found often useful and sometimes indispensable in the management of fever.

I have to-day given you this outline of some forms of inflammation of mucous membranes, omitting many things which I shall point out to you as the course advances. You perceive that the study of these diseases will be less easy and less beautiful than that of the serous inflammations, but it will not be the less necessary; for the mucous membranes have more ample relations with the functions of the body than any other tissue.

